# LRRFIP2 negatively regulates NLRP3 inflammasome activation in macrophages by promoting Flightless-I-mediated caspase-1 inhibition

**DOI:** 10.1038/ncomms3075

**Published:** 2013-08-14

**Authors:** Jing Jin, Qian Yu, Chaofeng Han, Xiang Hu, Sheng Xu, Qingqing Wang, Jianli Wang, Nan Li, Xuetao Cao

**Affiliations:** 1 Institute of Immunology, Zhejiang University School of Medicine, 866 Yu-Hang-Tang Road, Hangzhou 310058, China; 2 National Key Laboratory of Medical Immunology & Institute of Immunology, Second Military Medical University, 800 Xiangyin Road, Shanghai 200433, China; 3 National Key Laboratory of Medical Molecular Biology & Department of Immunology, Chinese Academy of Medical Sciences, 9 Dongdan Santiao, Beijing 100005, China; 4 These authors contributed equally to this work

## Abstract

The NLRP3 inflammasome is the most characterized inflammasome activated by cellular infection or stress, which is responsible for the maturation of proinflammatory cytokines IL-1β and IL-18. The precise molecular mechanism for negative regulation of NLRP3 inflammasome activation needs to be further defined. Here we identify leucine-rich repeat Fli-I-interacting protein 2 (LRRFIP2) as an NLRP3-associated protein and an inhibitor for NLRP3 inflammasome activation. LRRFIP2 binds to NLRP3 via its N terminus upon NLRP3 inflammasome activation, and also interacts with Flightless-I, a pseudosubstrate of caspase-1, via its Coil motif. Knockdown of Flightless-I significantly promotes NLRP3 inflammasome activation. LRRFIP2 enhances the interaction between Flightless-I and caspase-1, facilitating the inhibitory effect of Flightless-I on caspase-1 activation. Furthermore, silencing of Flightless-I abrogates the inhibitory effect of LRRFIP2 on NLRP3 inflammasome. These data demonstrate that LRRFIP2 inhibits NLRP3 inflammasome activation by recruiting the caspase-1 inhibitor Flightless-I, thus outlining a new mechanism for negative regulation of NLRP3 inflammasome.

## Abstract (web summary)

Inflammasomes promote the maturation of inflammatory cytokines in response to signals associated with damage and infection, but it remains unclear how these signals are attenuated. Here, the authors show that the NLRP3 inflammasome is inhibited by LRRFIP2 through recruitment of the protein Flightless I.

## Graphical abstract

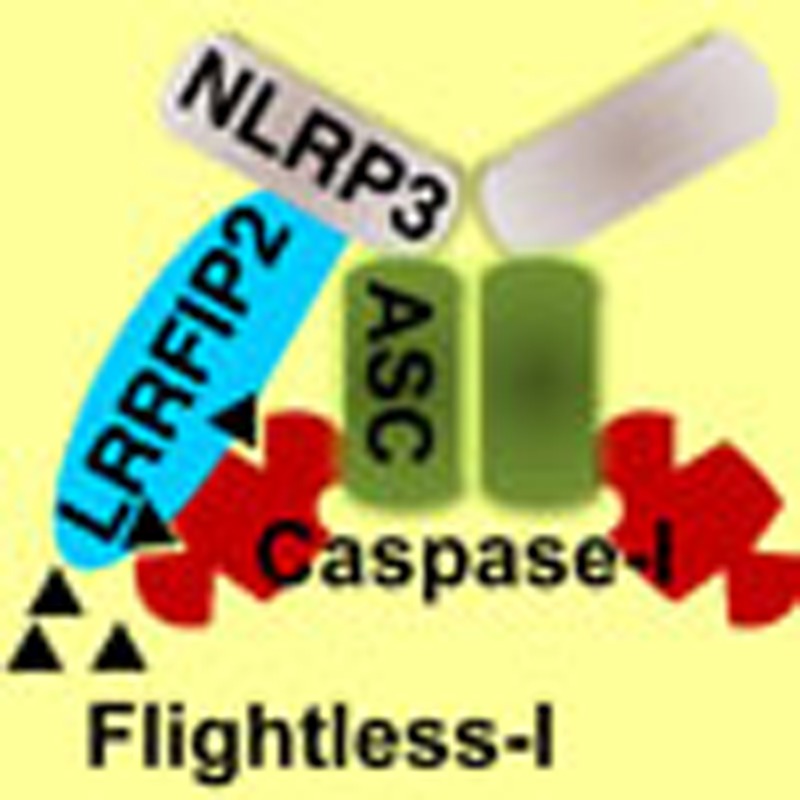

Inflammasomes are multiprotein complexes and serve as platforms for the activation of the cysteine protease caspase-1, which leads to the processing and secretion of the proinflammatory cytokines interleukin (IL)-1β (IL-1β) and IL-18^1^,^2^,^3^. Several NLR family members are reported to be capable of forming inflammasomes in response to their specific stimulators. The NLRP3 inflammasome is currently the most studied inflammasome which consists of the NLRP3 scaffold, the apoptotic speck protein containing a caspase recruitment domain (ASC) adaptor and caspase-1^4^,^5^,^6^,^7^. It has been associated with many kinds of diseases including autoimmune disorders, atherosclerosis, type 2 diabetes, gout and obesity^8^. Up to now, much attention has been paid for the identification of molecules to promote activation of NLRP3 inflammasome. However, the mechanisms for negative regulation of NLRP3 inflammasome activation are still poorly understood^9^.

To identify the potential regulator of the NLRP3 inflammasome activation, we screened for proteins that might interact with NLRP3 by co-immunoprecipitation (Co-IP) and liquid chromatography coupled with tandem mass spectrometry^10^,^11^, and identified a candidate protein, leucine-rich repeat Fli-I-interacting protein 2 (LRRFIP2). As a partner of Flightless-I, LRRFIP2 is widely expressed in many tissues including lung, liver, brain and muscle^12^. LRRFIP2 exhibits 41% sequence homology with LRRFIP1 (or Flap-1, Fli-I LRR-associated protein 1)^13^. LRRFIP1 was originally identified as a protein that interacts with the mammalian homologue of *Drosophila* flightless I (Fli-I), a member of the gelsolin family that is important for actin organization during *Drosophila* embryogenesis and myogenesis^14^. LRRFIP1 positively regulates thrombus formation by working as a component of the platelet cytoskeleton where it interacts with the actin-remodeling proteins Flightless-1^15^. We have reported LRRFIP1 as a cytosolic nucleic-acid sensor, which mediates the production of type I interferon induced by vesicular stomatitis virus and *Listeria monocytogenes* in macrophages via a β-catenin-dependent pathway^16^. Except the coil–coil domain conserved within LRRFIP1 and LRRFIP2, which has been predicted to serve as the potential interaction motif for Flightless-I-LRRFIPs^12^, little analysis has been performed on LRRFIP2 functional domain. LRRFIP2 has been revealed as an activator of Wnt by interacting with Dvl to activate β-catenin/LEF/TCF-dependent transcriptional activity^17^. Moreover, LRRFIP2 has also been reported as a positive regulator of TLR4 signalling by competitively disrupting the interaction between MyD88 and Flightless-I at a very early stage of TLR agonist stimulation^13^,^18^. However, the role of LRRFIP2 in inflammasome activation and regulation remains unknown.

In this paper, we describe a new mechanism by which LRRFIP2 negatively regulates NLRP3 inflammasome activity. LRRFIP2 could bind both NLRP3 and Flightless-1 by its N terminal and coiled motif, respectively. Knockdown of Flightless-I significantly promotes NLRP3 inflammasome activation. NLRP3 inflammasome activation signals triggered the binding of LRRFIP2 with NLRP3, which promotes Flightless-I to interact with NLRP3 inflammasome component caspase-1 and then suppresses the activity of caspase-1. Furthermore, *in vivo*-delivered small interfering RNA (siRNA) oligonucleotides dampening the expression of LRRFIP2 increase the Alum-induced production of IL-1β and subsequent inflammatory responses. Therefore, LRRFIP2 is a negative regulator of NLRP3 inflammasome activation by promoting inhibition of caspase-1 via Flightless-I, adding insight to the tight regulation of inflammasomes.

## Results

### LRRFIP2 inhibits NLRP3 inflammasome activation

**Figure 1:**
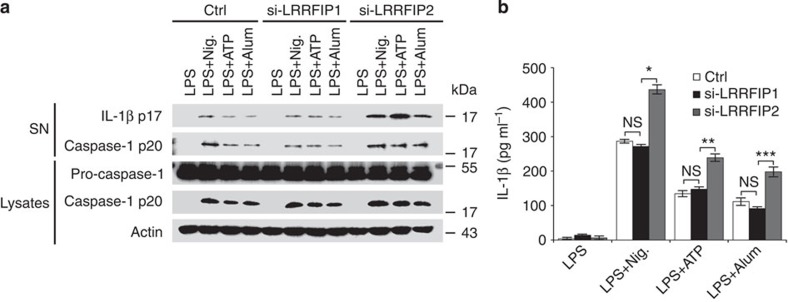
LRRFIP2 inhibits NLRP3 inflammasome activity in macrophages. (**a**) Immunoblot of the IL-1β, the pro-caspase-1 and cleaved caspase-1 in the supernatants (SNs) or cell lysates of LRRFIP2-silenced mouse primary macrophages, primed with LPS, and then stimulated with Nigericin (Nig.), ATP or Alum. The β-actin was also detected as loading control (Ctrl). (**b**) ELISA of IL-1β in SNs from macrophages silenced of LRRFIP2, incubated with LPS overnight, and followed by stimulation with Nig., ATP or Alum (*n*=3–6). *P=0.0347; **P=0.0351; ***P=0.0287 (two-tailed Student’s *t*-test). Data shown are representative of three separate experiments (**a**) or are means±s.e.m. of three independent experiments (**b**).

To identify potential regulators of the NLRP3 inflammasome, we first incubated a specific anti-NLRP3 antibody with total cell lysates of mouse macrophages with NLRP3 inflammasome activation (primed by lipopolysaccharide (LPS) and then stimulated with ATP). The co-immunoprecipitated complex was separated by SDS–polyacrylamide gel electrophoresis, and the specific fragments were analysed by liquid chromatography coupled with tandem mass spectrometry. Two independent experiments yielded 30 putative NLRP3-associated proteins candidates altogether. LRRFIP2 was the only protein identified from both two independent co-IP/MS experiments. Then, we investigated the biological function of LRRFIP2 in NLRP3 inflammasome activation. LRRFIP2 was efficiently down regulated in mouse primary peritoneal macrophages with specific interfering RNA, and the expression of NLRP3, ASC and caspase-1 was also not affected by LRRFIP2 silencing (Supplementary Fig. S1 and Fig. 1a). After knockdown of LRRFIP2, NLRP3 inflammasome activation was strengthened, as more cleaved caspase-1 was detected in LRRFIP2-silenced macrophages treated by NLRP3 inflammasome activator such as Nigericin, ATP or Alum (Fig. 1a). However, silencing LRRFIP1 had no influence on IL-1β secretion or inflammasome activation (Fig. 1a,b and Supplementary Fig. S2), suggesting that though similarity existed between LRRFIP1 and LRRFIP2, they had different roles in the regulation of NLRP3 inflammasome activation. Furthermore, IL-1β secretion was also significantly increased in macrophages silenced of LRRFIP2 (Fig. 1a,b). However, similar IL-1β mRNA expression level (Supplementary Fig. S3) was found in LRRFIP2-silenced and control siRNA-transfected macrophages, ruling out a general defect in macrophage responsiveness. We also detected the effect of LRRFIP2 on other inflammasome activation such as NLRC4 and AIM2 inflammasomes. However, the NLRC4 activator Flagellin-induced or AIM2 activator Poly (dA:dT)-induced IL-1β production was comparable between control and LRRFIP2-silenced macrophages (Supplementary Fig. S4). Therefore, these results indicated that LRRFIP2 inhibited IL-1β production posttranscriptionally by specifically inhibiting the NLRP3 inflammasome activation.

### LRRFIP2 interacts with NLRP3 through its N-terminal motif

**Figure 2:**
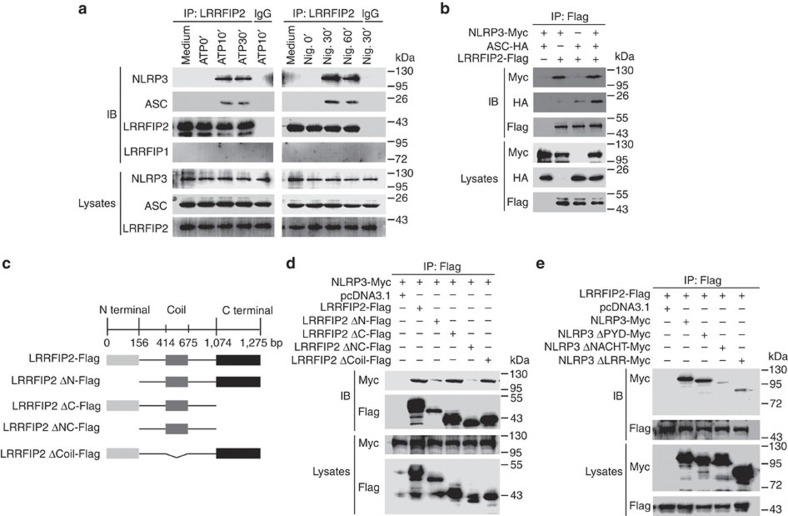
LRRFIP2 interacts with NLRP3 via its N-terminal motif. (**a**) Macrophages were primed with LPS and treated with ATP, Nigericin (Nig.) for the indicated times, immunoprecipitated with anti-LRRFIP2, and then immunobloted using the indicated antibodies. (**b**) 293T cells were co-transfected with expression plasmids for NLRP3-Myc, ASC-HA and LRRFIP2-Flag as indicated, immunoprecipitated with anti-Flag, and immunobloted using indicated antibodies. (**c**) LRRFIP2-truncated mutant vectors were constructed. (**d**) 293T cells were co-transfected with Myc-tagged NLRP3 and Flag-tagged LRRFIP2 truncated mutants, immunoprecipitated with anti-Flag and immunobloted using indicated antibodies. (**e**) 293T cells were co-transfected with Flag-tagged LRRFIP2 and Myc-tagged NLRP3 truncated mutants, immunoprecipitated with anti-Flag and immunobloted using indicated antibodies. Results are representative of three separate experiments with similar results. SN, supernatant.

Then, we identified the expression pattern of LRRFIP2 in macrophages (Supplementary Fig. S5, bottom). LPS robustly upregulated LRRFIP2 expression after 8 h. Although LRRFIP2 expression could be significantly induced by LPS, it did not bind to NLRP3 in macrophages treated with LPS only (Supplementary Fig. S5). Next, we investigated whether LRRFIP2 could indeed interact with NLRP3 after inflammasome activation. Immunoprecipitation assay showed that LRRFIP2 co-precipitated with NLRP3 and other components 10 min after ATP stimuli and 30 min after Nigericin stimuli (Fig. 2a). Therefore, LRRFIP2 could interact with the NLRP3 inflammasome complex very rapidly.

To investigate how LRRFIP2 interacted with NLRP3 inflammasome, we expressed LRRFIP2 with NLRP3, and ASCrespectively, in 293T cells. LRRFIP2 could significantly immunoprecipitate with NLRP3, and mildly immunoprecipitate with ASC (Fig. 2b). Furthermore, additional expression of NLRP3 promoted the interaction between ASC and LRRFIP2, while additional expression of ASC did not promote the interaction between NLRP3 and LRRFIP2 (Fig. 2b), suggesting that LRRFIP2 may indirectly interact with ASC through NLRP3.

To investigate which domain of LRRFIP2 is responsible for its interaction with NLRP3, four truncated mutants were constructed: LRRFIP2 ΔN-Flag lacking N terminal, LRRFIP2 ΔC-Flag lacking C terminal, LRRFIP2 ΔNC-Flag lacking both N and C terminals, and LRRFIP2 ΔCoil-Flag lacking Coil motif (Fig. 2c). Co-IP experiments with these truncated mutants showed that LRRFIP2 ΔN-Flag and LRRFIP2 ΔNC-Flag lost the ability to bind NLRP3, while LRRFIP2 ΔC-Flag and LRRFIP2 ΔCoil-Flag did not (Fig. 2d). These results indicated that LRRFIP2 interacted with NLRP3 via its N-terminal motif in response to NLRP3 inflammasome stimulations. However, both LRRFIP1 and LRRFIP1 ΔN-Flag failed to bind NLRP3 (Supplementary Fig. S6), suggesting the specificity of N terminal from LRRFIP2. Three truncated mutants of NLRP3 were also constructed. Co-IP experiments with these truncated mutants showed that NLRP3 ΔLRR-Myc and NLRP3 ΔNACHT-Myc lost the ability to bind LRRFIP2, while NLRP3 ΔPYD-Myc did not (Fig. 2e). So both the LRR and the NACHT domain of NLRP3 are necessary for the NLRP3–LRRFIP2 interaction (Fig. 2e).

### Motifs of LRRFIP2 required to inhibit NLRP3 inflammasome

**Figure 3:**
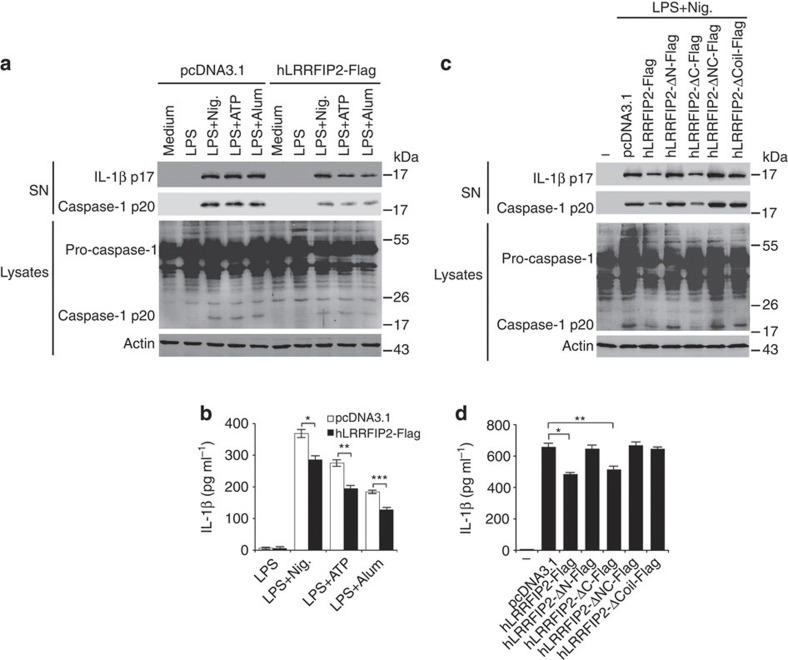
N-terminal and Coil motif are required for inhibitory effect of LRRFIP2 on NLRP3 inflammasome activation in THP-1 cells. (**a**,**b**) Phorbol myristate acetate (PMA)-pretreated THP-1 cells were transfected with human LRRFIP2 (hLRRFIP2-Flag), incubated with LPS overnight, and followed by stimulation with Nigericin (Nig.), ATP or Alum. Then the IL-1β, the pro-caspase-1, and cleaved caspase-1 in the supernatants or the cell lysates were analysed by immunoblot (**a**) and the IL-1β in the supernatants was also measured by ELISA (**b**) (*n*=3–6). *P=0.0395; **P=0.0327; ***P=0.0285 (two-tailed Student’s *t*-test). (**c**,**d**) PMA-pretreated THP-1 cells were transfected with hLRRFIP2 or its truncated mutants, incubated with LPS overnight and then treated with Nig. The IL-1β, the pro-caspase-1, and cleaved caspase-1 in the supernatants or the cell lysates were analysed by immunoblot (**c**), and IL-1β in cell culture supernatants was also measured by ELISA (**d**) (*n*=3–6). *P=0.0337; **P=0.0345 (two-tailed Student’s *t*-test). Data shown are representative of three separate experiments (**a**,**c**) or are means±s.e.m. of three independent experiments (**b**,**d**). Ctrl, control.

To confirm the inhibitory effect of LRRFIP2 on NLRP3 inflammasome activation in human, we overexpressed LRRFIP2 (hLRRFIP2-Flag) in phorbol myristate acetate-activated THP-1 cells and then stimulated with Nigericin, ATP or Alum. We found hLRRFIP2-Flag-transfected THP-1 cells produced less cleaved caspase-1 (Fig. 3a) and less IL-1β (Fig. 3a,b) than the control group, demonstrating that LRRFIP2 also inhibited NLRP3 inflammasome activity in human macrophages. Then, we constructed the truncated mutants of hLRRFIP2 as that in mice to learn which domains were necessary for its inhibitory function. Wild-type hLRRFIP2 as well as C-terminal loss mutants inhibited NLRP3 inflammasome activity, yet mutants with N-terminal deletion or Coil motif deletion failed to inhibit caspase-1 activation (Fig. 3c) and IL-1β production (Fig. 3c,d). These data indicated that besides the N terminal, which is responsible for its interaction with NLRP3, the Coil motifs of LRRFIP2 were also crucial for its inhibitory function on NLRP3 inflammasome activation.

### LRRFIP2 promotes Flightless-I interaction with caspase-1

**Figure 4:**
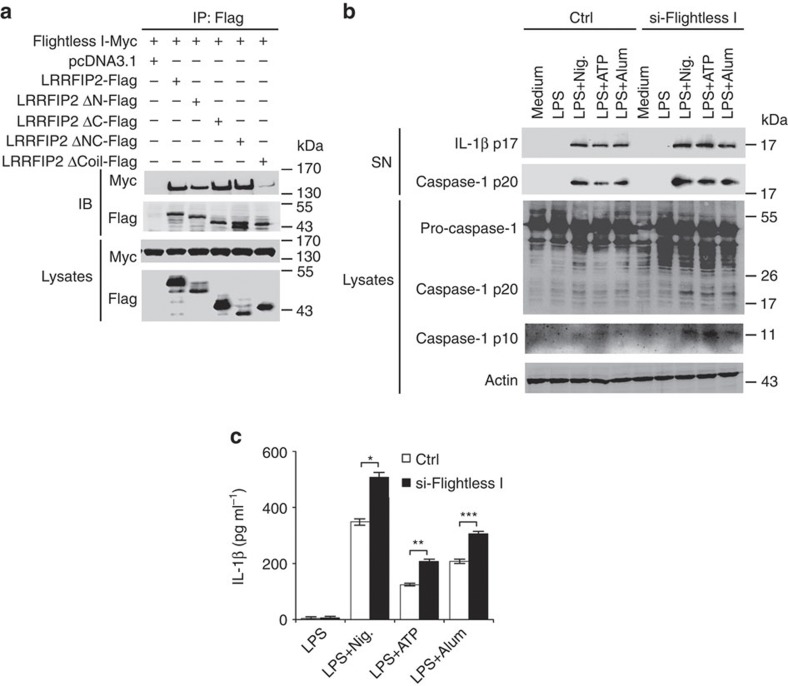
Flightless-I interacts with LRRFIP2 and inhibits NLRP3 inflammasome activation. (**a**) 293T cells were co-transfected with Myc-tagged Flightless-I and Flag-tagged LRRFIP2 or its truncated mutants, immunoprecipitated with anti-Flag and immunobloted using indicated antibodies. (**b**) Immunoblot analysis of the IL-1β, the pro-caspase-1, and cleaved caspase-1 in the supernatants or the cell lysates of macrophages silenced of Flightless I, primed with LPS and then treated with Nigericin (Nig.), ATP or Alum. (**c**) ELISA of IL-1β in supernatants from macrophages silenced of Flightless I, incubated with LPS overnight, and treated with Nig., ATP or Alum (*n*=3–6). *P=0.0311; **P=0.0263; ***P=0.0223 (two-tailed Student’s *t*-test). Data shown are representative of three separate experiments (**a**,**b**) or are means±s.e.m. of three independent experiments (**c**). Ctrl, control.

As the Coil motif was indispensable for the inhibitory effect of LRRFIP2, and LRRFIP2 could interact with Flightless-I via its Coil motif^12^, as Fig. 4a shows, we speculate that Flightless-I might participate in the negative regulation of NLRP3 inflammasome activity by LRRFIP2. We also found Flightless I is upregulated by LPS after 12 h (Supplementary Fig. S7a). Co-IP experiments in 293T confirmed the binding of Flightless-1 with caspase-1 (Supplementary Fig. S7b). Flightless-I knockdown significantly increased caspase-1 cleavage (Fig. 4b) and IL-1β secretion in LPS-primed macrophages treated by Nigericin, ATP or Alum (Fig. 4b,c). We also detected the effect of Flightless-I on the regulation of other inflammasome activation. Flightless-I knockdown significantly increased IL-1β secretion in LPS-primed macrophages transfected by Flagellin or Poly (dA:dT) (Supplementary Fig. S7c). These data suggested that Flightless I is a general inhibitor of the inflammasomes, consistent with previous reports that Flightless-I inhibits caspase-1 activity as a pseudosubstrate^19^.

**Figure 5:**
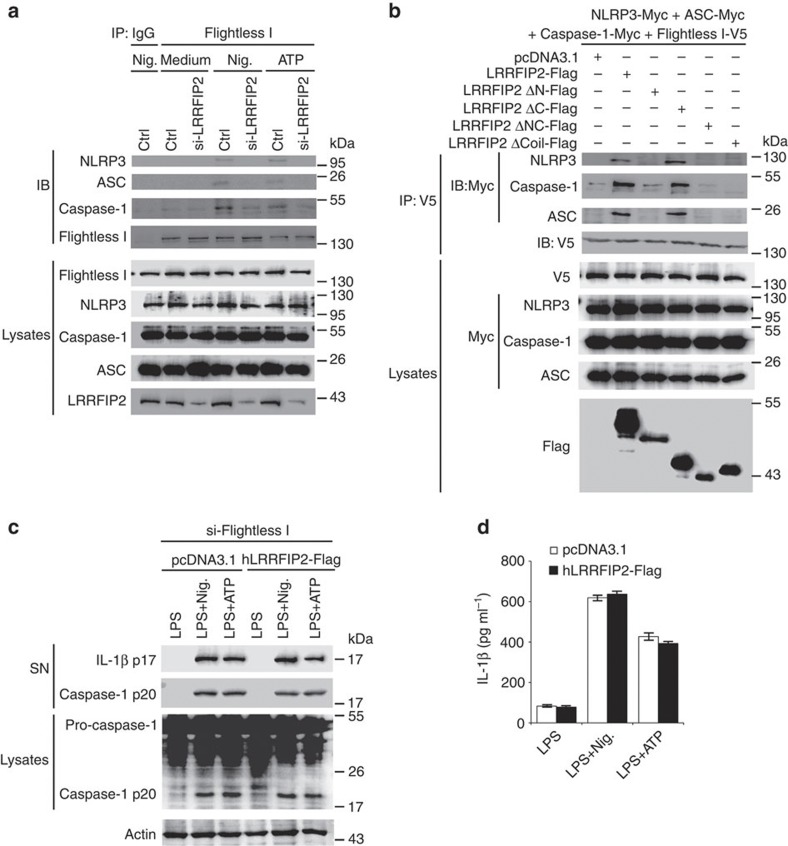
LRRFIP2 facilitates Flightless-I interaction with caspase-1 and inhibits NLRP3 inflammasome in macrophages. (**a**) Macrophages silenced of LRRFIP2 were primed with LPS and treated with Nigericin (Nig.) or ATP, immunoprecipitated with anti-Flightless-I and then immunobloted using indicated antibodies. (**b**) 293T cells were co-transfected with Myc-tagged NLRP3, ASC and caspase-1, V5-tagged Flightless-I and Flag-tagged LRRFIP2 mutants, immunoprecipitated with anti-V5 and immunobloted using indicated antibodies. (**c**,**d**) Flightless-I-silenced THP-1 cells were transfected with hLRRFIP2 truncated mutants, primed with LPS and treated with Nig. or ATP. Then the IL-1β, the pro-caspase-1, and cleaved caspase-1 in the supernatants or the cell lysates were analysed by immunoblot (**c**) and the IL-1β in cell supernatants was measured by ELISA (**d**) (*n*=3). Data shown are representative of three separate experiments (**a**–**c**) or are means±s.e.m. of independent experiments (**d**). Ctrl, control.

We further investigated whether LRRFIP2 negatively regulated NLRP3 inflammasome via Flightless-I in mouse primary peritoneal macrophages. SiRNA knocking down of LRRFIP2 impaired the interaction between Flightless-I and NLRP3 inflammasome induced by Nigericin or ATP (Fig. 5a), indicating that LRRFIP2 could promote Flightless-I to bind to NLRP3 inflammasome. Then we coexpressed NLRP3 inflammasome components and Flightless-I with LRRFIP2 or its truncated mutants in 293T cells. Only LRRFIP2 mutants containing both N-terminal and Coil motif strengthened the interaction between Flightless-I and NLRP3 inflammasome components while other mutants failed (Fig. 5b), suggesting an intermediate role of LRRFIP2 in the interaction between Flightless I and NLRP3 inflammasome. Furthermore, knockdown of endogenous Flightless-I abrogated the inhibitory effect of LRRFIP2 on caspase-1 activation (Fig. 5c) and IL-1β production (Fig. 5c,d). As Flightless-I was identified to be a pseudosubstrate, which is cleaved by caspase-1, we also transfected the 293T cells with caspase-1 and LRRFIP2 expression vectors and found no cleavage of LRRFIP2 (Supplementary Fig. S8), suggesting LRRFIP2 is not a pseudosubstrate of caspase-1. These results suggested a non-redundant role of Flightless-I in LRRFIP2 function, and LRRFIP2 regulate NLRP3 inflammasome activation via facilitating Flightless-I-caspase-1 interaction.

### LRRFIP2 suppresses Alum-induced peritoneal inflammation

**Figure 6:**
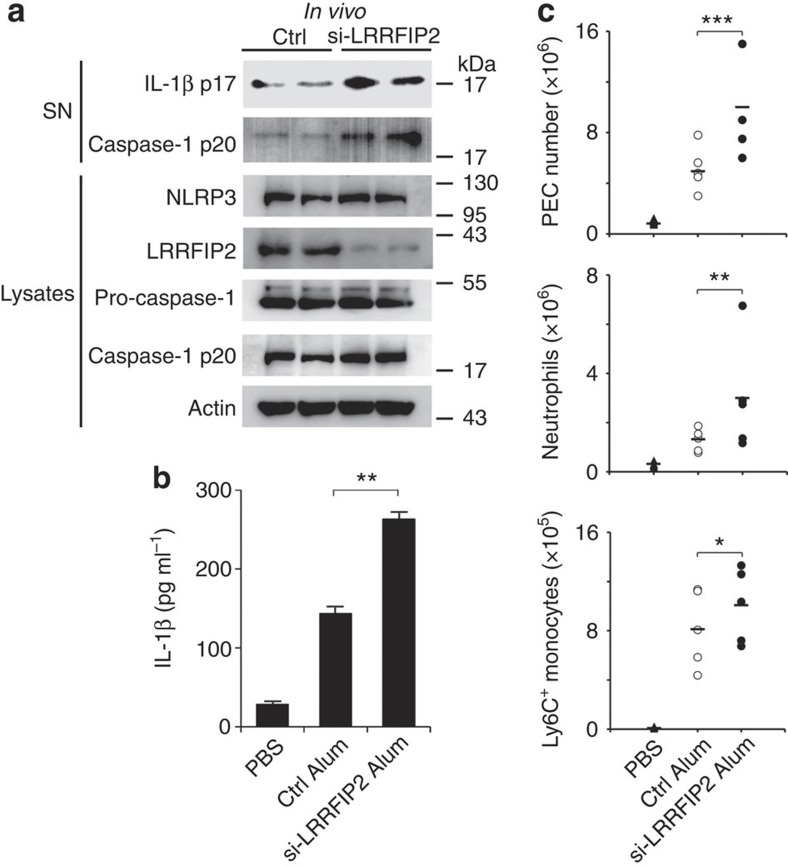
LRRFIP2 suppresses Alum-induced peritonitis *in vivo*. (**a**) Immunoblot analysis with indicated antibodies of PECs recovered 12 h after alum injection from LRRFIP2-silenced mice and control (Ctrl) mice (*n*=5–7). (**b**) IL-1β content in the lavage fluid 8 h after alum injection from LRRFIP2-silenced mice and Ctrl mice (*n*=5–7). **P=0.0074 (two-tailed Student’s *t*-test). (**c**) Inflammatory cell subset analysis by flow cytometry of PECs recovered 12 h after alum injection (*n*=5). *P=0.0282; **P=0.0029; ***P=0.0038 (analysis of variance). Data shown are representative of three separate experiments (**a**,**c**) or are means±s.e.m. of three independent experiments (**b**).

Given that LRRFIP2 inhibits NLRP3 inflammasome activation *in vitro*, we further demonstrated the biological effect of LRRFIP2 *in vivo* by knocking down LRRFIP2 in a mouse peritonitis model. The siRNA oligos complexed with *in vivo*-jetPEI, a linear polyethylenimine, significantly reduced the endogenous protein level of LRRFIP2 in the peritoneal cavity (Fig. 6a). Mice transfected with siRNA–jetPEI complex was stimulated by Alum to trigger peritonitis. Silencing of LRRFIP2 enhanced caspase-1 activation *in vivo* but didn’t affect the expression of NLRP3 and pro-caspase-1 in peritoneal exudates cells (PECs; Fig. 6a). IL-1β secretion in the lavage fluid was significantly increased by *in vivo* LRRFIP2 knockdown (Fig. 6a,b), which was in line with the observation *in vitro*. Alum-induced recruitment of inflammatory cells was then analysed by flow cytometry. The number of total PECs recruited upon Alum challenge was markedly increased in mice silencing of LRRFIP2 (Fig. 6c). Neutrophils and Ly6C^+^ monocytes recruitment were also significantly increased in the si-LRRFIP2 transfection group (Fig. 6c). These findings further demonstrated that LRRFIP2 can inhibit NLRP3 inflammasome activation and subsequent immune cell accumulation in mouse peritonitis *in vivo*.

## Discussion

Inflammasomes are involved in host response to a wide variety of damage- and pathogen-associated molecular patterns, mediating the cleaving of caspase-1 and the following processing of IL-1β. As IL-1β, IL-18 and pyroptotic death responses have the potential to damage the host, inflammasomes must be tightly regulated to avoid detrimental inflammatory responses. The over-activation of NLRP3 inflammasome resulted from NLRP3 gene mutation in human is highly correlated with some autoinflammatory syndromes^20^ and metabolic diseases^3^,^8^. Several strategies were employed by pathogens to block the inflammasome-mediated host defenses, for example, poxvirus protein M013 binds ASC and inhibits inflammasome activation^2^. The host itself also develops several strategies to control inflammasomes activity. Endogenous POPs such as POP1 and POP2 bind to the PYD of ASC and NLRs, respectively, and sequester them from the inflammasome complex^21^. Endogenous COPs, such as COP1 (pseudo-ICE), INCA, ICEBERG and caspase-12, which are hypothesized to function in a manner analogous to that of POPs, bind to CARD domain of caspase-1 and sequester it from inflammasome activation^2^. The Bcl-2 family members Bcl-2 and Bcl-X_L_, have been shown to specifically interact with NLRP1 to inhibit ATP binding and subsequent oligomerization^22^.

Here we identified a new negative regulator of NLRP3 inflammasome, LRRFIP2, after screening NLRP3-associated proteins via coimmunoprecipitation followed by mass spectrometry. Multiple lines of evidence implicate LRRFIP2 as a negative regulator of inflammasomes, which terminates excessive inflammatory responses through recruitment of Flightless-I: (a) dampening of LRRFIP2 promotes NLRP3 inflammasome-mediated caspase-1 cleavage and the following IL-1β production in mouse macrophages; (b) N-terminal and coil motif of LRRFIP2 are required for its inhibitory effect on NLRP3 inflammasome activation in THP-1 cells; (c) LRRFIP2 could bind to NLRP3 via its N terminal upon NLRP3 inflammasome activation and interact with Flightless-I via its Coil motif; (d) silencing of Flightless-I significantly abrogates the inhibitory function of LRRFIP2 on inflammasome activation; (e) silencing of LRRFIP2 *in vivo* potently increases the Alum-induced peritonitis. LRRFIP2 co-immunoprecipitated with NLRP3 quickly after ATP or Nigericin stimulation, representing an important regulatory strategy to limit the activity of inflammasomes. Through the instant activation of negative regulators, the immune system constantly strikes a balance between activation and inhibition to avoid detrimental and inappropriate inflammatory responses. This regulation pattern has been profoundly illustrated by previous research on the regulation of TLR-mediated immune responses^23^.

Caspase-1 is the central components of the inflammasomes. Several inhibitors of caspase-1 have been documented, such as the serpin protease inhibitor 9 (ref. 24) and CrmA, a cowpox virus-encoded inhibitor of caspase-1^25^. Recently, cellular inhibitor of apoptosis proteins was also reported to induce the non-degradative polyubiquitination of caspase-1, indicating caspase-1 as an important target for controlling inflammasomes activity^26^. As the executor of inflammasomes, most of previous studies focus on the direct inhibitors of caspase-1, however, little is known about how these inhibitors are linked to the inflammasomes complex. Here, we propose a model that LRRFIP2 inhibits NLRP3 inflammasome activation through facilitating the Flightless-I–caspase-1 interaction. Upon NLRP3 inflammasome activation, LRRFIP2 protein interacts with NLRP3 protein through its N-terminal motif. Meanwhile, LRRFIP2 could also interact with Flightless-I, the direct caspase-1 protease inhibitor, via its Coil motif. The LRRFIP2–Flightless-I interaction promotes Flightless-I to interact with caspase-1 and eventually inhibits NLRP3 inflammasome activity. Even so, the factors recruited to caspase-1 and their unexpected biological functions during the inflammasome quiescent state need further investigation.

Flightless-I, originally identified from a *Drosophila melanogaster* mutant^27^, contains a C-terminal gelsolin-like domain and belongs to the gelsolin superfamily^28^. Flightless-I has been found to inhibit caspase-1 activation as a pseudosubstrate in a manner similar to CrmA, which contains a caspase-1 substrate recognition sequence and functions as an inhibitory substrate^19^,^25^. Moreover, Flightless-I has an N-terminal leucine-rich repeat (LRR) protein–protein interaction domain that has many identified binding partners, including LRRFIPs, TRIP and CaMK-II^12^,^14^,^29^,^30^. The connection between LRRFIP2–Flightless-I interaction and Flightless-I-mediated caspase-1 inhibition we reported here have revealed the whole picture of Flightless-I interacting proteins. LRRFIP2 enhances the interaction between Flightless-I and NLRP3 inflammasome, which facilitates the inhibitory function of Flightless-I. Our results have uncovered a pathway crucial for suppression of NLRP3 inflammasome. The cooperation of LRRFIP2 and Flightless-I to inhibit caspase-1 activation also uncovered the orchestrated regulation of inflammasomes.

Overall, our findings demonstrate that NLRP3 inflammasome activation leads to interaction between NLRP3 and LRRFIP2, which facilitates Flightless-I-mediated caspase-1 inhibition. Moreover, we have described a new mechanism of inflammasome regulation by targeting its component caspase-1. This finding may provide potential targets for the design of anti-inflammatory therapies.

## Methods

### Mice and reagents

Female C57BL/6 mice (7–8 weeks) were obtained from SIPPR-BK Experimental Animal Co. (Shanghai, China). All animal experiments were undertaken in accordance with the National Institutes of Health Guide for the Care and Use of Laboratory Animals, with the approval of the Scientific Investigation Board of Second Military Medical University, Shanghai. The LPS, Nigericin, ATP and phorbol myristate acetate were from Sigma-Aldrich. Imject Alum (77161) was from Thermo Scientific. Flagellin and Poly (dA:dT) were from Invivogen.

### Antibodies

Anti-HA (ab9110), anti-Myc (Myc A.7), anti-V5 (ab9116), and anti-Flag (G-10) were from Abcam. Anti-mouse caspase-1 p10 (M-20), anti-LRRFIP2 (D-17), anti-ASC (N-15), anti-LRRFIP1 (34) and Anti-Flightless I (E-1) were from Santa Cruz. Anti-β-actin (AC-15) was from Sigma-Aldrich. Anti-mouse caspase-1 p45&p20 (Casper-1) and Anti-NLRP3 (Cryo-2) were from AdipoGen. The horseradish peroxidase-conjugated second antibodies (TrueBlot) were from eBioscience.

### Cell culture

Peritoneal macrophages were collected from mice 4 days after thioglycollate (BD, Sparks, MD) injection. Then peritoneal macrophages were cultured in endotoxin-free RPMI1640 with 10% (vol/vol) fetal bovine serum^31^. Human THP-1 and 293T cells were obtained from American Type Culture Collection and cultured in RPMI 1640 and DMEM, respectively, supplemented with 10% fetal bovine serum.

### Plasmids construction and transfection

Recombinant vectors encoding murine LRRFIP2 (NM_027742), NLRP3 (NM_145827), ASC (NM_023258), Caspase-1(NM_009807), FLI (NM_022009) and human LRRFIP2 (NM_017724) were constructed by PCR-based amplification of cDNA from macrophages or THP-1 cells, respectively, then were subcloned into the pcDNA3.1 eukaryotic expression vector (Invitrogen). Truncated mutants of LRRFIP2, LRRFIP1 and NLRP3 were obtained by PCR-based mutation and amplification of wild-type LRRFIP2 expression vectors. Plasmids were transiently transfected into 293T cells or THP-1 cells with jetPEI reagents according to the manufacturer’s instructions (Polyplus).

### RNA interference assay

siRNAs were synthesized as following: LRRFIP2: 5′-GAUGCCAAUAGACAAAUUAUU-3′, Flightless-I: 5′-AAACAAGAAUGAGC GGAAAUU-3′ and negative control: 5′-AATCAGTCACGTTAATGGTCG-3′. These siRNA duplexes were transfected into mouse peritoneal macrophages or THP-1 cells using INTERFERin (PolyPlus) according to the manufacturer’s standard protocol^32^.

### Measurement of cytokine production

Peritoneal macrophages or THP-1 cells (5 × 10^5^) were seeded in 24-well plates and cultured overnight. After priming with 100 ng ml^−1^ LPS for 12 h and stimulating with Nigericin (10 μM), ATP (1 mM) or Alum (200 μg ml^−1^), the supernatants were collected, and the concentrations of IL-1β were measured using mouse IL-1β or human IL-1β ELISA kits (R&D Systems), according to the manufacturer’s instruction.

### Immunoprecipitation and immunoblot analysis

The lysates of 293T cell were immunoprecipitated for 3 h at 4 °C with anti-Flag conjugated beads (Sigma-Aldrich) or anti-V5 conjugated beads (ab1229 Abcam). The lysates of primary mouse peritoneal macrophages were immunoprecipitated overnights at 4 °C with 2 μg ml^−1^ anti-LRRFIP2 or anti-Flightless I antibodies and protein A-agarose beads. After extensive washing with lysis buffer, the immunocomplexes and any non-covalently bound proteins were dissociated by boiling in 5 × loading buffer (Sangon, Shanghai) and subjected to SDS–polyacrylamide gel electrophoresis, followed by immunoblotting^11^,^33^. Cell culture supernatants were precipitated by the addition of an equal volume of methanol and 0.25 volumes of chloroform, then were vortexed and centrifuged for 10 min at 20,000 *g*. The upper phase was discarded and 500 ml methanol was added to the interphase. This mixture was centrifuged for 10 min at 20,000 *g* and the protein pellet was dried at 55 °C, resuspended and boiled for 5 min at 99 °C.

### *In vivo* siRNA transfection

*In vivo* siRNA delivery was carried using *in vivo*-jetPEI (Polyplus, CA) according to the manufacturer’s instructions. Generally, mice were intraperitoneally injected with transfection mixture consisting of siRNA dissolved in *in vivo*-jet PEI with the N/P ratio of 7 according to the instruction. The interfering efficacy was determined by western blot.

### *In vivo* peritonitis

For induction of peritonitis, mice were i.p. injected with 700 μg Alum (Thermo). For analysis of inflammatory cell subsets, mice were killed 12 h after alum injection and peritoneal cavities were washed with 6 ml of PBS. PECs were collected and analysed by flow cytometry^34^. For the analysis of IL-1β in the peritoneal cavity, 8 h after i.p. injection of Alum, peritoneal cavities were washed with cold PBS. Then the peritoneal fluids were concentrated for ELISA analysis with Amicon Ultra 10K from Millipore.

### Statistical analysis

All experiments were independently performed three times in triplicate. Results are given as means±s.e. Comparisons between two groups were performed using Student’s *t*-test or analysis of variance, with a value of *P*<0.05 considered to be statistically significant.

## Author contributions

X.C. designed research; J.J., Q.Y., C.H., X.H., S.X. and N.L. performed research; Q.W. and J.W. contributed the reagents; X.C., J.J. and Q.Y. analysed data and wrote the paper.

## Additional information

**How to cite this article:** Jin, J. *et al*. LRRFIP2 negatively regulates NLRP3 inflammasome activation in macrophages by promoting Flightless-I-mediated caspase-1 inhibition. *Nat. Commun.* 4:2075 doi: 10.1038/ncomms3075 (2013).

## Supplementary Material

Supplementary Information Supplementary Figures S1-S8
