# Supplementary material for: LRRFIP2 negatively regulates NLRP3 inflammasome activation in macrophages by promoting Flightless-I-mediated caspase-1 inhibition
Source: Nat Commun. 2013 Aug 14;4:2075. doi: 10.1038/ncomms3075 (PMC3753543; doi:10.1038/ncomms3075)
Supplement: Supplementary Information — Supplementary Figures S1-S8 [file ncomms3075-s1.pdf]

Supporting materials for

LRRFIP2 negatively regulates NLRP3 inflammasome activation in macrophages by promoting Flightless-I-mediated caspase-1 inhibition

Jing Jin, Qian Yu, Chaofeng Han, Xiang Hu, Sheng Xu, Qingqing Wang, Jianli Wang, Nan Li, and Xuetao Cao

This PDF file includes

Supplementary Figures S1-S8

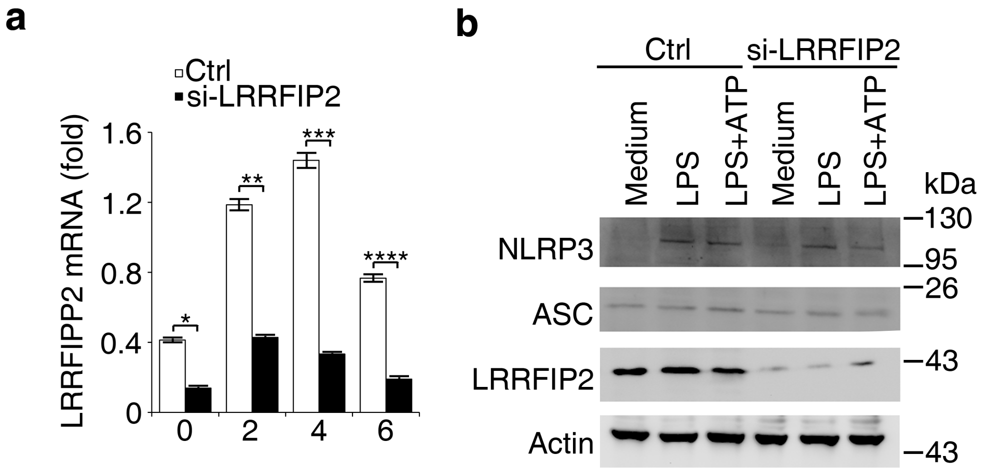

Supplementary Figure S1. Expression of NLRP3 and ASC after LRRFIP2 was silenced in macrophages.

(a) Mouse primary peritoneal macrophages silenced of LRRFIP2 were treated by LPS for the indicated times. Relative mRNA expression of LRRFIP2 was measured by quantitative PCR (n = 3). *P = 0.0021; **P = 0.0017; ***P = 0.0013; ****P = 0.0016; (two-tailed Student's t-test). (b) The protein level of LRRFIP2, NLRP3 and ASC in cell lysates of macrophages silenced of LRRFIP2, primed with LPS, and then stimulated with ATP were analyzed by immunoblot. Data shown are means±s.e.m. of 3 independent experiments (a) or are representative of three separate experiments (b).

Jin Jing, et al. Fig. S2

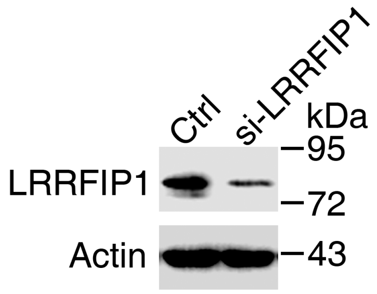

Supplementary Figure S2. Efficient silencing of LRRFIP1 in macrophages.

The protein level of LRRFIP1 in cell lysates of macrophages silenced of LRRFIP1, primed with LPS, was analyzed by immunoblot. Data shown are representative of three separate experiments.

Jin Jing, et al. Fig. S3

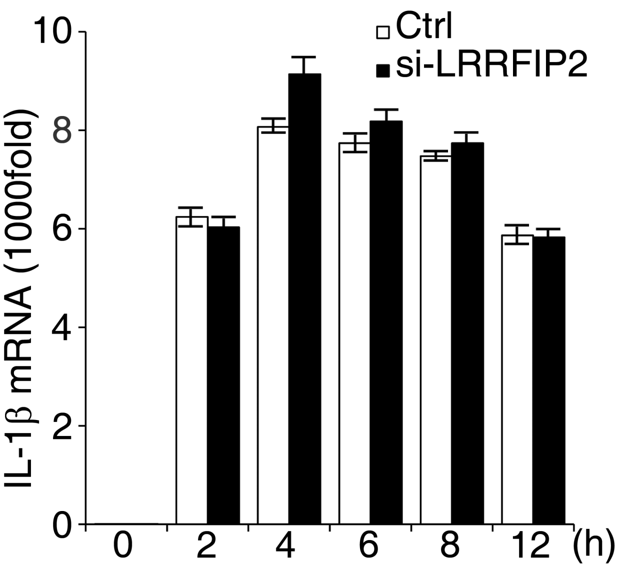

Supplementary Figure S3. Silencing of LRRFIP2 does not affect LPS-induced IL-1β transcription in macrophages.

Mouse primary peritoneal macrophages silenced of LRRFIP2 were treated with LPS for the indicated times. Relative mRNA expression of IL-1β was measured by quantitative PCR (n = 3). Data shown are means±s.e.m. of 3 independent experiments.

Jin Jing, et al. Fig. S4

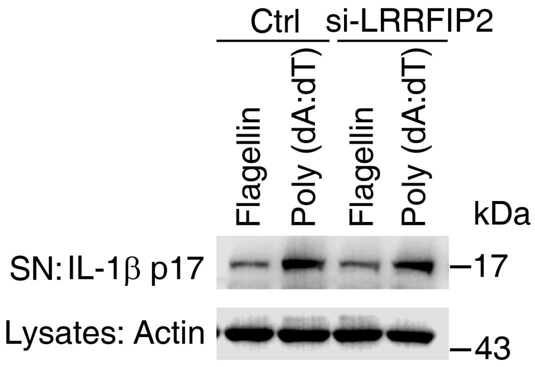

Supplementary Figure S4. Silencing of LRRFIP2 does not affect NLRC4 or AIM2 inflammasome induced IL-1β maturation in macrophages.

Immunoblot of the IL-1β in the supernatants (SN) of LRRFIP2-silenced macrophages, primed with LPS, and then transfected with Flagellin or Poly (dA:dT). The β-Actin was also detected as loading control. Data shown are representative of three separate experiments.

Jin Jing, et al. Fig. S5

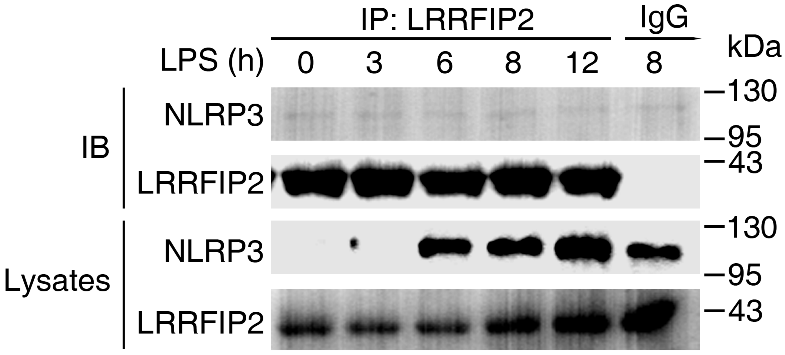

Supplementary Figure S5. LRRFIP2 doesn't interact with NLRP3 in macrophages stimulated with LPS.

Macrophages were primed with LPS for the indicated times, immunoprecipitated with anti-LRRFIP2, and then immunobloted using the indicated antibodies. Data shown are representative of three separate experiments.

Jin Jing, et al. Fig. S6

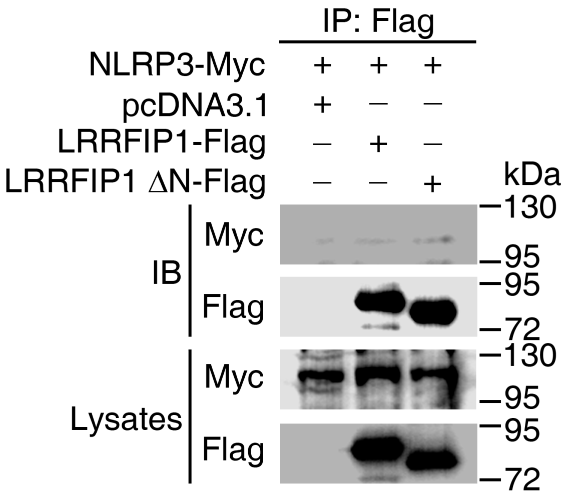

Supplementary Figure S6. LRRFIP1 doesn't interact with NLRP3 in 293T cells.

293T cells were co-transfected with Myc-tagged NLRP3 and Flag-tagged LRRFIP1 or LRRFIP1-ΔN, immunoprecipitated with anti-Flag, and immunobloted using the indicated antibodies. Data shown are representative of three separate experiments.

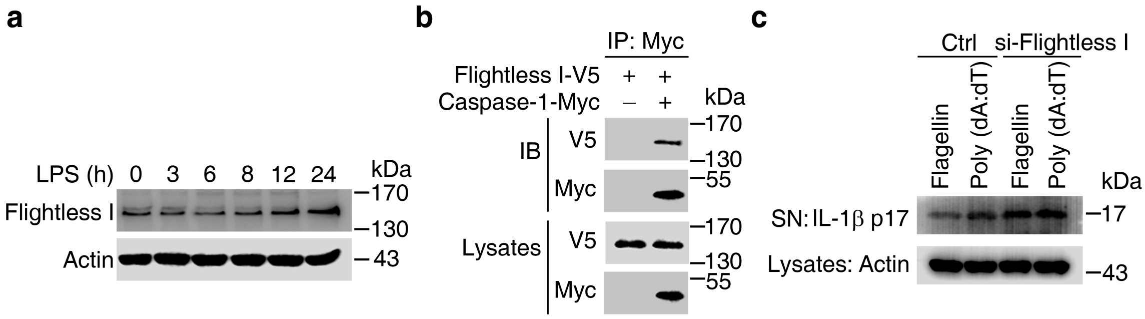

Supplementary Figure S7. Flightless I binds and inhibits caspase-1.

(a) Immunoblot of Flightless I in macrophages stimulated with LPS for the indicated times. (b) 293T cells were co-transfected with expression plasmids for Flightless I-V5, and caspase-1-Myc as indicated, immunoprecipitated with anti-Myc, and immunobloted using indicated antibodies. (c) Immunoblot of the IL-1β in the supernatants (SN) of Flightless-I-silenced macrophages, primed with LPS, and then transfected with Flagellin or Poly (dA:dT). Data shown are representative of three separate experiments.

Jin Jing, et al. Fig. S8

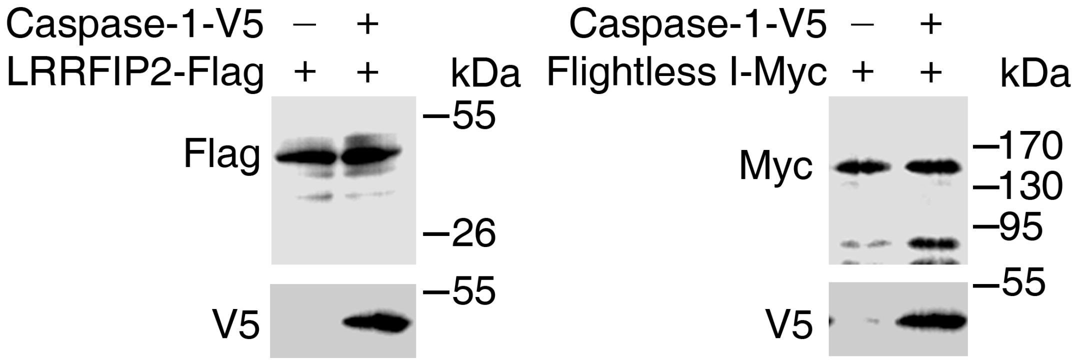

Supplementary Figure S8. LRRFIP2 is not cleaved by caspase-1 in 293T cells.

293T cells were co-transfected with expression plasmids for caspase-1-V5 with LRRFIP2-Flag or Flightless I-Myc as indicated, and immunobloted using indicated antibodies. Data shown are representative of three separate experiments.
